# Supplementary material for: Morphological and Biochemical Changes in the Mediterranean Cereal Cyst Nematode (Heterodera latipons) during Diapause
Source: Pathogens. 2024 Aug 2;13(8):656. doi: 10.3390/pathogens13080656 (PMC11357521; doi:10.3390/pathogens13080656)
Supplement: Supplementary file 1 [file pathogens-13-00656-s001.zip › pathogens-3094812-supplementary.pdf]

Supplementary Materials

Formatted

Figure S1

Photomicrographs of cyst and vulval cone of *Heterodera latipons* showing: A) cyst (whole female) and terminal cone region. B) vulval cone top showing strong underbridge with bi-trifurcate extremities. C) vulval cone showing vulval slit separating semi-fenestrae. D) vulval cone showing two semi-fenestrae with few scattered bullae.

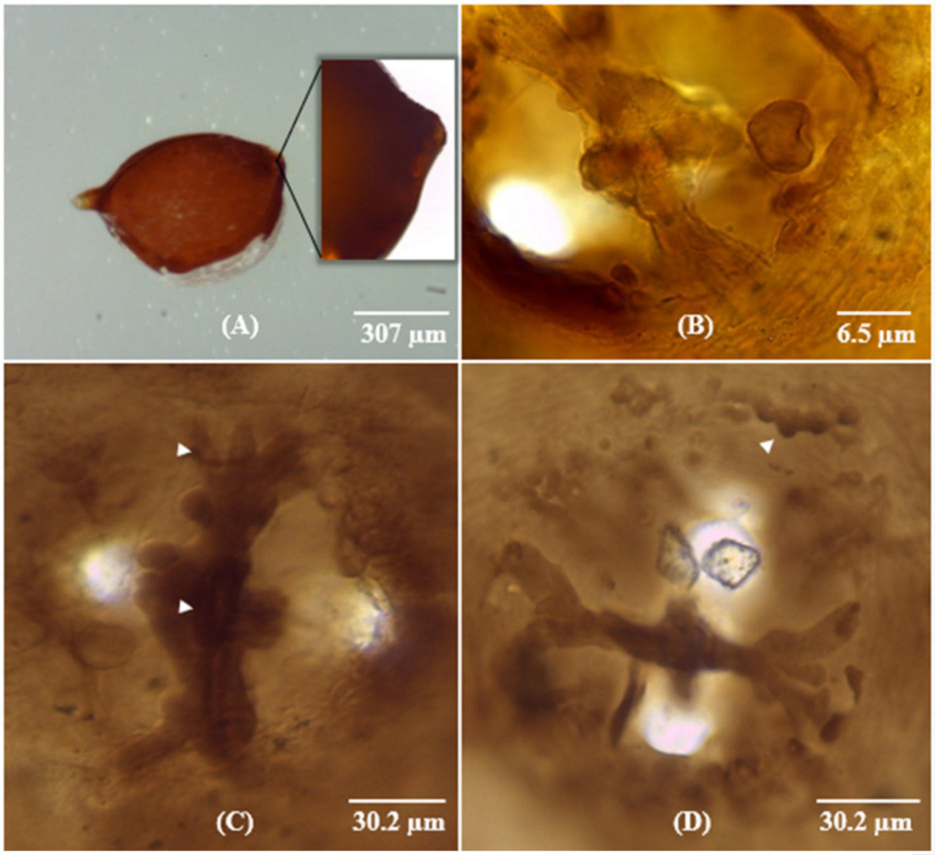

Figure S2

Photomicrographs of J2s and eggs of *Heterodera latipons* showing: A) whole juvenile B) anterior region showing lip region, strong stylet with basal knobs, dorsal esophageal gland opening (DEGO), and median bulb. C) pharyngeal region showing median bulb, ventral pharyngeal overlap and ~~ventrally overlapping esophagus and~~ an excretory pore (arrowed). D-1) tail region showing anus (arrowed). D-2) tail region showing hyaline terminus and a wrinkled tail. E1-2) coiled J2 inside eggshell.

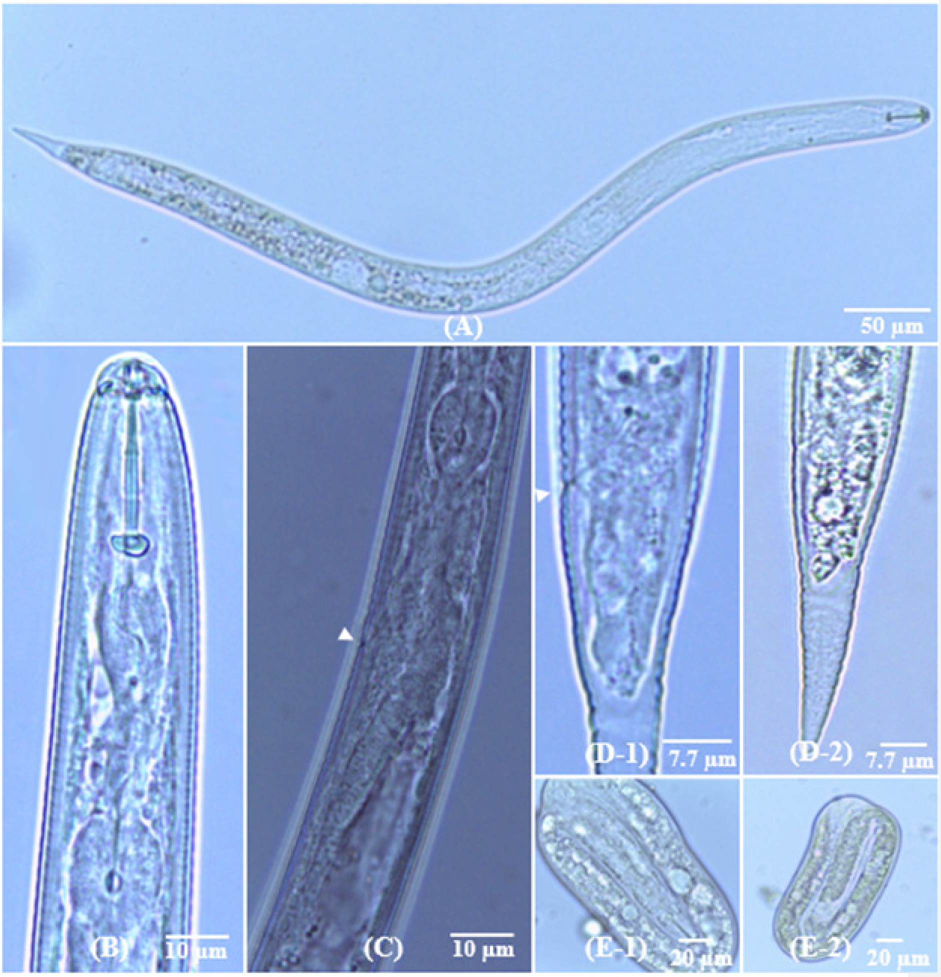

Figure S3

PCR products using *Heterodera latipons* DNA from the cysts that collected in October 2021 by using different 10 gene-specific primer pairs. Lane M: 50-bp DNA ladder (GeneDirex), lane 1: *Age*-1 gene, lane 2: *Daf*-2 gene, lane 3: *Daf*-7 gene, lane 4: *Daf*-11 gene, lane 5: *Daf*-18 gene, lane 6: *Daf*-21 gene, lane 7: *Tps*-(1+2) genes, lane 8: *Tre*-1, *Tre*-2, and *Tre*-3 genes, lane 9: *Tre*-4 gene, lane 10: *Tre*-5 gene.

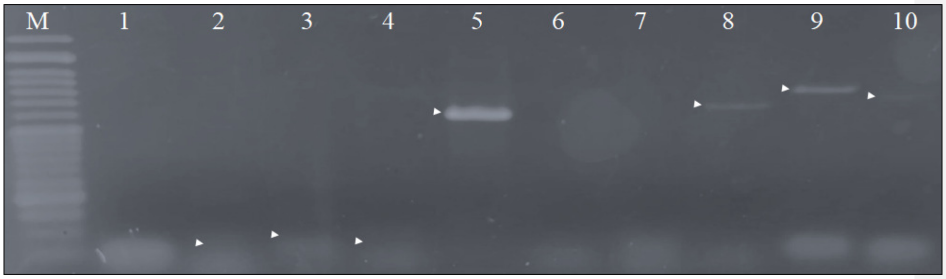

Figure S4

Maximum and minimum temperature in Madaba, Jordan during June 2021-October 2021 (https://www.accuweather.com).

Field Code Changed

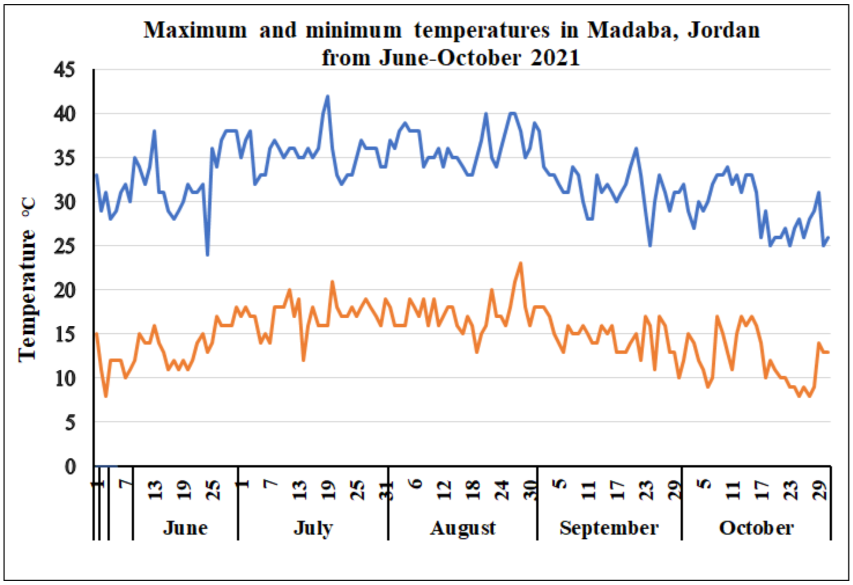

Table S1. EST contigs of *Heterodera avenae* for ten diapause genes after BLASTx with plant parasitic nematodes.

No.	Gene name	*Caenorhabditis elegans* accession no. *	Plant parasitic nematode		Contig (*Heterodera avenae*)
			Organism	Accession no.	
1	***Age*-1**	NM_064061	*Aphelenchus avenae*	KAH7724728	sra_data-1_trimmed_contig_4727
2	***Daf*-2**	AF012437	*Meloidogyne enterolobii*	CAD2170559	sra_data_contig_9672
3	***Daf*-7**	NM_064864	*Aphelenchus avenae*	KAH7731921	sra_data-1_trimmed_contig_3384
4	***Daf*-11**	NM_073559	*Meloidogyne enterolobii*	CAD2147153	sra_data_contig_13702
5	***Daf*-18**	NM_067525	*Aphelenchus avenae*	KAH7725458	sra_data-1_trimmed_contig_2500
6	***Daf*-21**	GU441459	*Meloidogyne enterolobii*	CAD2189708	sra_data_contig_2159
7	***Tps*-1**	AJ512332	*Aphelenchus avenae*	Q5K2C1	sra_data-1_trimmed_contig_386
8	***Tre*-1**	AJ512337	*Meloidogyne enterolobii*	CAD2175231	sra_data-1_trimmed_contig_23355
9	***Tre*-4**	AJ512338	*Meloidogyne enterolobii*	CAD2192164	sra_data-1_trimmed_contig_861
10	***Tre*-5**	AJ512339	*Aphelenchus avenae*	KAH7694133	sra_data_contig_14348

* Source: [22,31].

Table S2. Primer pairs served for detection of *Heterodera latipons* diapause genes.

No.	Gene name	Primer name *	Sequence (5'-3')	Amplicon length (bp)
**1**	*Age*-1	H.l_F01	CTGACCTCACTCAGTTCCAT	511
		H.l_R01	TGTAGATGTGCTTGTTCAGC	
**2**	*Daf*-2	H.l_F02	GAAAGTGAGTACGGCTATGG	463
		H.l_R02	AGGTGTCTTGAGAAGCAAAC	
**3**	*Daf*-7	H.l_F03	GCATCAAGATTCTGTTCGTC	647
		H.l_R03	GTCTCGGGAAATTTTTGGTC	
**4**	*Daf*-11	H.l_F04	GCAACAAAAGATTGAGCAGA	754
		H.l_R04	CCCTGTGAGTGTGATGATAG	
**5**	*Daf*-18	H.l_F05	ATTTGTCATCCTCATCTTCG	528
		H.l_R05	GTGGAAAGGAAAGAGGTTTT	
**6**	*Daf*-21	H.l_F06	GAAGTTGTGCAAAGTCATCA	458
		H.l_R06	CAATCTTCTCCGCAATAGTC	
**7**	*Tps*-1 and *Tps*-2	H.l_F07	CTAAGAGAGAGGCTTGATAGC	799
		H.l_R07	TATCTTTTGTCGGAGGATCT	
**8**	*Tre*-1, *Tre*-2, and *Tre*-3	H.l_F08	AACAGACGAGAATGATCACC	439
		H.l_R08	TCGATGAGCATATGAGACAA	
**9**	*Tre*-4	H.l_F09	CTGTTCAGTCAAATGATCGC	636
		H.l_R09	CACTTTTTGGCACTTTTCCT	
**10**	*Tre*-5	H.l_F10	GAGTATTTTGAAGCCACGC	600
		H.l_R10	AATGGAGTGGTAACATCGC	

* F, forward; R, reverse.

Table S3. Standardized canonical discriminant function coefficients for *Heterodera latipons* (this study) and different populations of *Heterodera* spp. based on 23 morphometrical characters of J2s and cysts.

Trait	Function									
	1	2	3	4	5	6	7	8	9	10
**Second stage juvenile**										
Body length (L)	-.560	.549	-1.015	-.986	.093	.036	-.072	.333	.380	-.283
Midbody width (W)	.452	.258	.291	-.049	.684	.368	.072	-.312	-.077	-.255
a= L/W	.030	.225	.494	.423	.763	-.316	-.041	.580	-.149	.122
Distance (anterior end to end of median bulb)	.589	-.277	.526	-.454	.041	.383	.781	-.274	-.412	-.397
b^m= L/distance from lip to end of median bulb	.514	-1.500	.224	-.242	-.373	.252	.675	-.113	-.816	-.805
Tail length	.940	.576	-.040	1.124	-.544	.632	-.175	-.030	-.069	.276
c= L/tail length	.010	.012	-.277	.247	-.497	-.366	-.237	-.445	.691	.569
Body width at anus	-.965	-.180	-.114	.547	-.081	-.542	.424	.189	.828	.428
Tail length/body width at anus	-1.190	.047	-.320	.087	.141	-.195	.223	-.496	.581	.076
Hyaline length	.065	-.837	.119	-.436	-.012	.306	-.209	-.239	-.196	1.009
Stylet length	.320	.226	.086	-.099	-.496	.026	-.037	.011	.175	.691
Hyaline length/stylet length	-.418	.441	.254	.020	-.162	-.136	.639	.473	.196	-.343
Distance (dorsal gland duct opening to stylet base)	-.301	.387	.235	.142	.281	-.056	.108	-.285	.116	-.255
Head width	.122	.259	.046	-.243	.049	-.386	.167	-.474	-.811	-.022
Head height	-.061	.693	-.296	-.641	-.473	.415	-.365	.512	.093	-.292
**Cyst**										
Fenestral length	.046	.422	-.599	.332	-.276	-.304	.212	.294	-.484	.090
Fenestral width	.140	-.128	1.655	.316	.096	.546	-.096	-.173	.180	-.267

Semi-fenestral length	-.144	-.555	-1.180	.083	.834	-.298	-.819	.483	-.003	.274
Distance of vulval slit to semi-fenestra	1.409	-.060	.044	.127	.115	.173	.252	.099	-.060	-.038

Table S4. Result of the BLASTx and BLASTn from the obtained sequences of *daf-* genes and *tre* genes.

No.	**Gene name**	**BLASTx**	**BLASTn**
**1**	*Daf-2*	NSSF*	NSSF
**2**	*Daf-7*	NSSF	94% to *Digitaria exilis* (Accession LR792834)
**3**	*Daf-11*	NSSF	NSSF
**4**	*Daf-18*	NSSF	87% to *H. glycines* (Accession CP049712)
**5**	*Tre-1, Tre-2,* and *Tre-3*	NSSF	NSSF
**6**	*Tre-4*	NSSF	87% to *Radix natalensis* (Accession HQ283257)
**7**	*Tre-5*	See **Table 4**	85% to *H. glycines* (Accession CP049709)

* NSSF = No significant similarity found

Table S5. Temperature and humidity of Madaba, Jordan (June-October 2021) (https://www.accuweather.com; https://www.timeanddate.com).

Month (2021)	Humidity		Precipitation	Temperature	
	Average	Range		Maximum (range) °C	Minimum (range) °C
**June**	44%	11-80%	0	24-38	8-17
**July**	43%	9-80%	0	32-40	12-21
**August**	41%	9-88%	0	33-40	13-23
**September**	53%	14-94%	0	28-38	10-18
**October**	51%	15-94%	0	25-34	8-17
